# Maternal Smoking During Pregnancy and Childhood Growth Trajectory: A Random Effects Regression Analysis

**DOI:** 10.2188/jea.JE20110033

**Published:** 2012-03-05

**Authors:** Kohta Suzuki, Naoki Kondo, Miri Sato, Taichiro Tanaka, Daisuke Ando, Zentaro Yamagata

**Affiliations:** 1 Center for Birth Cohort Studies, Interdisciplinary Graduate School of Medicine and Engineering, University of Yamanashi, Yamanashi, Japan; 2 Department of Health Sciences, Interdisciplinary Graduate School of Medicine and Engineering, University of Yamanashi, Yamanashi, Japan; 3 Department of Physical Education, National Defense Academy, Kanagawa, Japan

**Keywords:** body mass index, childhood growth, gender, multi-level analysis, pregnancy, smoking

## Abstract

**Background:**

Although maternal smoking during pregnancy has been reported to have an effect on childhood overweight/obesity, the impact of maternal smoking on the trajectory of the body mass of their offspring is not very clear. Previously, we investigated this effect by using a fixed-effect model. However, this analysis was limited because it rounded and categorized the age of the children. Therefore, we used a random-effects hierarchical linear regression model in the present study.

**Methods:**

The study population comprised children born between 1 April 1991 and 31 March 1999 in Koshu City, Japan and their mothers. Maternal smoking during early pregnancy was the exposure studied. The body mass index (BMI) z-score trajectory of children born to smoking and non-smoking mothers, by gender, was used as the outcome. We modeled BMI trajectory using a 2-level random intercept and slope regression.

**Results:**

The participating mothers delivered 1619 babies during the study period. For male children, there was very strong evidence that the effect of age in months on the increase in BMI z-score was enhanced by maternal smoking during pregnancy (*P* < 0.0001). In contrast, for female children, there was only weak evidence for an interaction between age in months and maternal smoking during pregnancy (*P* = 0.054), which suggests that the effect of maternal smoking during pregnancy on the early-life BMI trajectory of offspring differed by gender.

**Conclusions:**

These results may be valuable for exploring the mechanism of fetal programming and might therefore be clinically important.

## INTRODUCTION

In recent years, the obesity pandemic has become a major global public health issue.^1^ In Japan, the prevalence of obesity in adults is still relatively low,^2^^,^^3^ but the prevalence of overweight and obesity has steadily increased in both genders among people younger than 50 years.^4^ In addition, the prevalence of childhood obesity has been steadily increasing since the 1980s.^5^ Moreover, gender differences have become apparent with regard to childhood obesity in Japan. While the prevalence in boys in 2007 was approximately twice (9%–10%) that of the reference data in 1979–81, for each age group, the prevalence in girls was less than that of boys, at approximately 8%.^5^ This difference is thought to reflect the recent decrease in mean body mass index (BMI) among young women.^4^

Recently, the percentage of women who smoke during pregnancy has also increased (5.6% in 1990 to 10.0% in 2000) in Japan,^6^ a trend opposite to that in other industrialized countries.^7^ Further, some studies have observed an association between maternal smoking during pregnancy and childhood obesity.^8^^–^^11^ Children with mothers who smoked during pregnancy tended to show a rapid increase in body weight, and to be overweight, in infancy and childhood.^12^ Further, it has been suggested that there may be a gender difference in the effect of maternal smoking during pregnancy, since gender differences in the prevalence of childhood obesity have been observed with the recent increase in the maternal smoking rate in Japan.

Previously, we reported gender differences in the effect of maternal smoking during pregnancy on childhood growth. We found that, among children born to smoking mothers, the BMI z-score trajectory at each subsequent check-up age was higher for boys than for girls. Among girls, the only difference in the slope of the trajectory was observed between age 3 and 5 years.^13^ However, after age 7 to 8 years, no significant gender difference was observed.^13^ A limitation of that analysis was that age (in years) was rounded and used as year dummy variables, which significantly decreased measurement precision. Consequently, the effect of age on childhood BMI z-scores could only be roughly estimated. The random-effects hierarchical linear regression model is able to use age in months as the time variable. Hence, it is a more appropriate method for correctly estimating the effect of age on the association between maternal smoking during pregnancy and childhood growth.

Thus, this study aimed to clarify the effect of maternal smoking during pregnancy on the trajectory of childhood BMI z-scores in both genders by using a more methodologically sound approach, ie, a random-effects hierarchical linear regression that models the exact age in months and also maximizes the information used. This approach allows for variability in the number of data points for BMI measurement.

## METHODS

### Study design

Children born between 1 April 1991 and 31 March 1999 in Koshu City, Yamanashi Prefecture, Japan and their mothers were included in this study. These children and mothers also participated in an ongoing prospective cohort study in rural Japan, known as Project Koshu (formerly, Project Enzan). Previous studies have described this project in detail.^10^^,^^11^^,^^13^^,^^14^ The dataset for the present study was similar to that of a previous study.^13^

This study was approved by the Ethical Review Board of the University of Yamanashi, School of Medicine and was conducted in accordance with the Guidelines Concerning Epidemiological Research (Ministry of Education, Culture, Sports, Science and Technology and Ministry of Health, Labour and Welfare, Japan). In addition, the Koshu City administrative office collaborated with the authors of this study.

### Data collection

The mothers were asked to complete a self-report questionnaire on their lifestyle habits during early pregnancy, including smoking status; these questionnaires were administered at the time of pregnancy registration. The Maternal and Child Health Handbook was used to collect data on the birth height and weight of children. Moreover, the height and weight of children aged 3 and 5 years and those of children in grades 2 and 4 of elementary schools (ie, aged 7–8 and 9–10 years, respectively) were measured during a medical check-up. A stadiometer (unit: 0–1 cm) and conventional weighing scales (unit: 100 g) were used to measure height and body weight, respectively. BMI was calculated according to World Health Organization (WHO) standards (body weight [kg]/height [m^2^]).

### Statistical analyses

An individual growth analysis method (SAS Proc Mixed) was used to compare BMI z-score trajectories born to smoking and non-smoking mothers of boys and girls. Adopting the approach of Twisk,^15^ we used 2-level random intercepts and a random slopes model to explore the association between maternal smoking during pregnancy and childhood BMI z-score trajectory. In particular, we modeled a random intercept that allowed the baseline BMI z-score to randomly vary across individuals and random slopes for age in months, maternal smoking during pregnancy, and the age-smoking interaction. We set an unstructured covariance structure for these random effects. Fixed and random parameters were estimated by restricting the maximum likelihood algorithm.

In this analysis, we used individual BMI data that were recorded at birth and at 1 or more times after the children were 3 years of age. Individual BMI z-scores, which were based on WHO standards, were used to adjust the differences in BMI for each month of age within the same age group.^16^^,^^17^ All statistical analyses were performed using SAS version 9.2 (SAS Institute Inc., Cary, NC, USA).

## RESULTS

The participating mothers delivered 1619 babies during the study period. Birth weight and anthropometric data were collected from 1603 (at birth, 99.0%), 1358 (age 3, 83.9%), 1248 (age 5, 77.1%), 1270 (age 7–8, 78.4%), and 1274 (age 9–10, 78.7%) of these children.

For boys, the BMI z-score increased as age in months increased (*P* < 0.0001). However, there was no evidence of a relationship between BMI z-score trajectory and maternal smoking during pregnancy (*P* = 0.7). Regarding the interaction term between age in months and maternal smoking, there was very strong evidence that maternal smoking during pregnancy enhanced the effect of age in months on the increase in BMI z-scores (*P* < 0.0001; Table, Figure).

**Figure.**
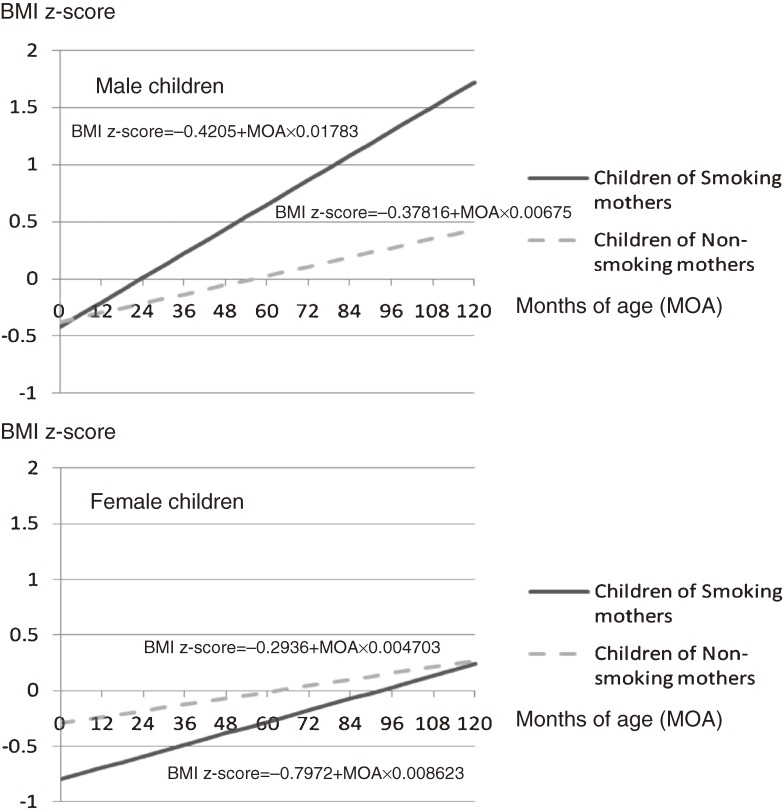
Childhood body mass index (BMI) z-score trajectories calculated by individual growth analysis based on random intercept and random slope models, as shown, for smoking and non-smoking mothers

**Table.** Solution for fixed effects in the random intercept and random slope model for months of age of the children (MOA), smoking status of their mother, and interaction between MOA and smoking status of their mother in the Koshu Project, 1991–2008

Factor	Male children				Female children			
	
	Estimate	Standard error	*t* value	*P*-value	Estimate	Standard error	*t* value	*P*-value
Intercept	−0.42	0.12	−3.6	0.0003	−0.80	0.14	−5.61	<0.0001
Months of age of children (MOA)	0.02	0.002	8.08	<0.0001	0.01	0.002	4.35	<0.0001
Non-smoking mother	0.04	0.12	0.35	0.73	0.50	0.15	3.44	0.0006
MOA * Non-smoking mother	−0.01	0.002	−4.88	<0.0001	−0.004	0.002	−1.92	0.054

For girls, there was very strong evidence that BMI z-score also increased as age in months increased (*P* < 0.0001). In addition, there was very strong evidence for a relationship between BMI z-score trajectory and maternal smoking during pregnancy (*P* = 0.0006). However, there was only a weak interaction between age in months and maternal smoking during pregnancy (Table, Figure).

## DISCUSSION

The present study confirmed our previous findings (ie, that the effect of maternal smoking during pregnancy on the early-life BMI trajectory of offspring differed by gender) but used an analytic method with greater validity and precision. As boys grew up, they were more likely than girls to be affected by maternal smoking during pregnancy. Among boys, although BMI z-score significantly increased as age in months increased, the effect of maternal smoking during pregnancy was not significant, which could enhance the effect of age in months on the increase in BMI z-score. In contrast, among girls, the coefficients of both age in months and the interaction term between age in months and maternal smoking were smaller than those in boys. Consequently, the effect of maternal smoking on BMI z-score in girls was smaller than that in boys. These results were consistent with those of our previous study, which used a fixed effect model.^13^

Some studies have suggested that girls are less vulnerable to adverse environmental factors such as exposure to smoking.^18^ Moreover, Smith et al have shown that prenatal nicotine exposure results in higher testosterone levels in rat fetuses,^19^ and Blouin et al have suggested that androgens play an important role in regulating body fat distribution.^20^ Our results appear to be consistent with these biological explanations. In contrast, Wisniewski and Chernausek have suggested that girls are more susceptible to environmental factors associated with obesity.^21^ However, they did not include a Japanese population in their study. Thus, it may be necessary to conduct further studies of the effects of ethnic differences.

In conclusion, smoking by pregnant mothers increases childhood weight gain, especially in boys. This result may be valuable for exploring the mechanism of fetal programming and might thus be clinically important. For example, it is important to conduct further studies on gender differences in fetal programming to clarify the mechanism of obesity-related diseases such as type 2 diabetes.
